# Deep brain stimulation of the posterior subthalamic area and the thalamus in patients with essential tremor: study protocol for a randomized controlled pilot trial

**DOI:** 10.1186/s13063-016-1599-3

**Published:** 2016-09-29

**Authors:** Michael T. Barbe, Jeremy Franklin, Daria Kraus, Paul Reker, Till A. Dembek, Niels Allert, Jochen Wirths, Jürgen Voges, Lars Timmermann, Veerle Visser-Vandewalle

**Affiliations:** 1 Department of Neurology, University Hospital Cologne, Kerpener Str. 62, 50937 Cologne, Germany; 2 Cognitive Neuroscience (INM3), Institute of Neuroscience and Medicine, Research Centre Jülich, Jülich, Germany; 3 Institute of Medical Statistics, Informatics and Epidemiology, University Cologne, Cologne, Germany; 4 Clinical Trials Centre Cologne, Cologne, Germany; 5 Rehabilitation Center Godeshoehe, Bonn, Germany; 6 Department of Stereotactic and Functional Neurosurgery, University Hospital Cologne, Kerpener Str. 62, D-50937 Cologne, Germany; 7 Department of Stereotactic Neurosurgery, University Hospital Magdeburg, Magdeburg, Germany; 8 Leibniz Institute for Neurobiology (LIN), Magdeburg, Germany

**Keywords:** Deep brain stimulation (DBS), Ventral intermediate nucleus of the thalamus (VIM), Posterior subthalamic nucleus (PSA), Essential tremor (ET), Zona incerta (Zi), Stimulation-induced side effects, Constant current stimulation, Crossover study

## Abstract

**Background:**

Deep brain stimulation (DBS) of the ventral intermediate nucleus (VIM) of the thalamus is effective in medication refractory essential tremor (ET). In recent years, evidence has accumulated that the region ventral to the VIM, the posterior subthalamic area (PSA), might be an equally or even more effective target for electrode implantation. However, this evidence is primarily based on case series, cross-sectional observations, and retrospective data.

**Methods/design:**

A prospective crossover pilot study investigating the effects of PSA stimulation in medication refractory ET patients was designed. In this study, bilateral electrodes are implanted such that at least one of the electrode contacts is located in the PSA and VIM, respectively. This implantation approach allows (1) a prospective double-blind investigation of the effects of PSA stimulation compared to baseline, as well as (2) a crossover comparison between VIM and PSA stimulation with respect to tremor suppression and side effect profiles.

**Discussion:**

The results of this double-blinded, prospective study will allow a better understanding of the effects and side effects of PSA compared to VIM-DBS in patients with ET.

**Trial registration:**

German Clinical Trials Register: DRKS00004235. Registered on 4 July 2012.

## Background

Essential tremor (ET) is the most common movement disorder with a prevalence of approximately 5 % in a population of 65 years of age and older [1]. ET clinically presents as a bilateral, largely symmetrical postural or kinetic tremor involving hands and forearms [2] and sometimes even the legs, the trunk, or the head and voice. Depending on the severity of the symptoms, ET can lead to significant impairment of activities of daily living [3] and may reduce quality of life [4].

Medical treatment of ET is often unsatisfactory or limited by side effects in up to 50 % of patients with ET [5]. Deep brain stimulation (DBS) is an effective and safe treatment option for pharmacologically resistant ET [6]. The target of choice hitherto has been the ventral intermediate nucleus (VIM) of the thalamus (VIM-DBS) [7]. However, habituation of tremor suppression [8] and stimulation-induced long-term side effects such as gait ataxia [9] or stimulation-induced dysarthria [10] may limit the net benefit of DBS in this target area and reduce individual perception of quality of life.

In recent years, the posterior subthalamic area (PSA), including the prelemniscal radiation and the zona incerta, emerged as a potential new anatomical target to treat ET [11–14] (for a review see Xie et al. [15]). Some studies found a superior effect of PSA stimulation in comparison to VIM stimulation when the same stimulation parameters were used [16–18]. Few studies compared the effect of the two target areas under the best clinical settings: whereas Sandvik et al. found more than half of the best clinical contacts in the PSA [19], Chang et al. compared VIM, PSA, and simultaneous VIM and PSA stimulation and observed no differences in the overall outcome of tremor suppression [20]. Another retrospective, non-randomized study reported 70 % hand tremor reduction in the VIM group and 89 % in the PSA group [21]. Few studies investigated the side effects of PSA stimulation, which can mainly be stimulation-induced dysarthria, gait ataxia, or limb paresthesia contralateral to the stimulation side [22, 23].

So far, to our knowledge, no controlled prospective studies on PSA stimulation in patients with ET with comparison to VIM stimulation are available. To this end, we designed a prospective, controlled pilot study investigating the outcome of PSA stimulation in ET patients. Bilateral electrodes are implanted such that VIM and PSA can be stimulated with different contacts of the same lead to allow a direct comparison of the two target areas in a randomized, double-blinded crossover design. This approach allows comparison of tremor reduction, quality of life, and side effects during PSA stimulation with baseline, as well as with VIM stimulation.

### Study questions

#### Study question 1

Study question 1 asks: Does PSA stimulation reduce tremor severity under PSA-DBS compared to baseline as measured by the Tremor Rating Scale (TRS) [24]? Furthermore, we expect an increase of general and disease-specific quality of life postoperatively (SF-36 [25] and Quality of life in Essential Tremor Questionnaire, QUEST [4]) and a certain degree of stimulation-induced side effects under PSA stimulation such as dysarthria [10] or gait ataxia [9].

#### Study question 2

Study question 2 asks: Does PSA stimulation reduce tremor severity compared to VIM stimulation measured by the TRS in a double-blinded crossover? Although the reports comparing PSA- and VIM-DBS are partially conflicting, we hypothesize a superiority of PSA over VIM stimulation with regard to tremor suppression. As described earlier, this superiority could be eroded due to side effects that might occur under PSA stimulation (so that ideal stimulation parameters cannot be set due to side effects) or due to application of more current in the VIM, thus equalizing the effect on tremor [17]. Due to the overall lack of studies comparing quality of life and DBS-induced side effects between VIM- and/or PSA-DBS, the study remains exploratory, since we were unable to create specific hypotheses for the occurrence of side effects in both regions.

## Methods

### Study design

**Fig. 1:**
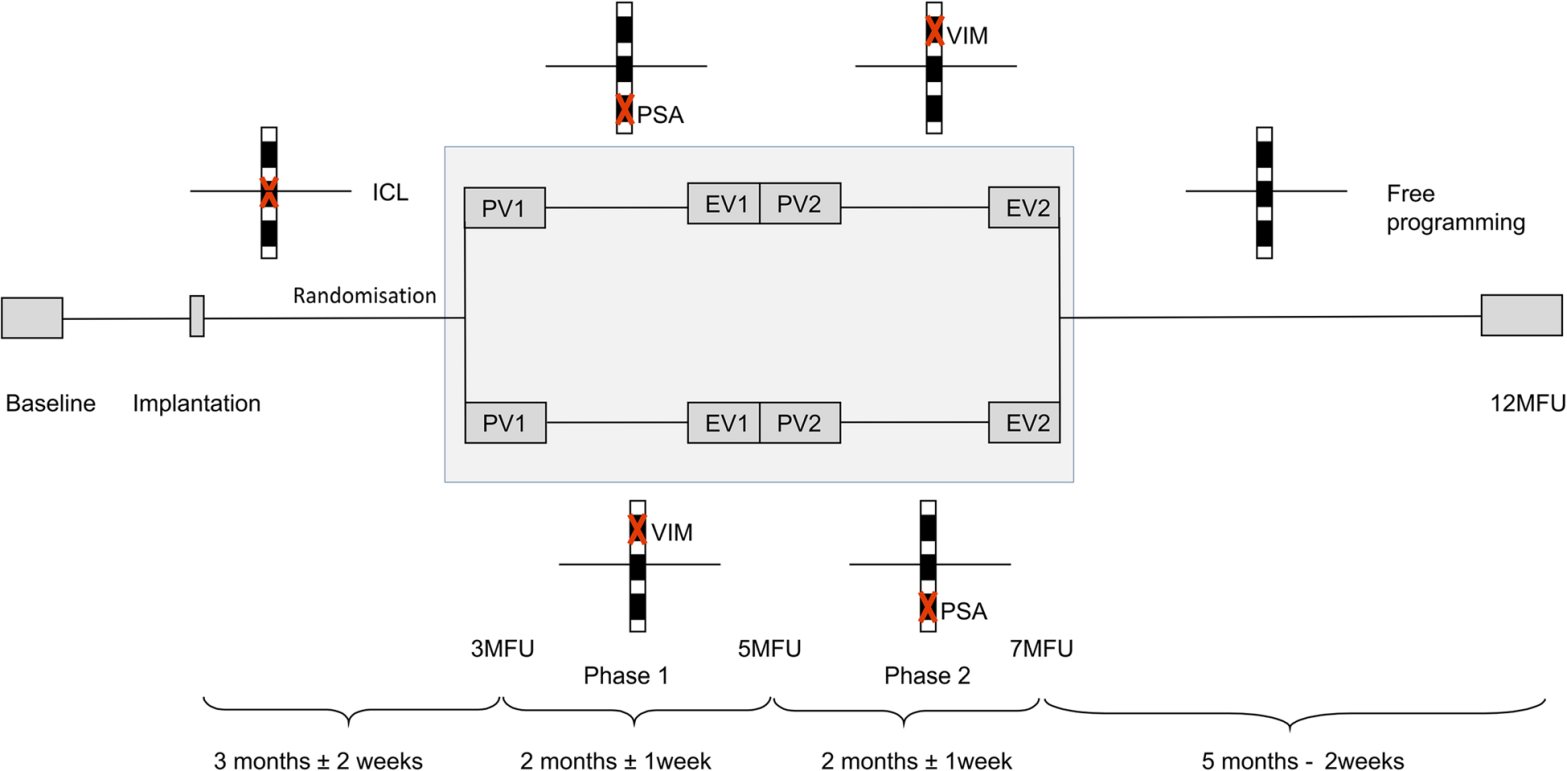
After enrollment and baseline evaluation, patients will be bilaterally implanted with DBS electrodes in the VIM and PSA. The electrodes are placed such that one contact is located on the intercommissural line (*ICL*), the neighboring ventral contact in the PSA, and the neighboring dorsal contact in the VIM. After implantation, the neutral contact on the ICL is activated until 3 months follow-up (*3MFU*). At 3MFU patients are randomized into a blinded crossover phase for 4 months (*gray box*). Depending on randomization, patients receive stimulation in the sequence PSA-VIM or VIM-PSA. Each phase of the crossover starts with a programming visit (*PV*) and ends with an evaluation visit (*EV*). After the blinded crossover phase, the stimulation contacts can be freely chosen until 12MFU

The study design is illustrated in Fig. 1. After baseline evaluation, patients are implanted as described below (see study procedure). Three months post-implantation, patients enter a double-blinded crossover phase with VIM- and PSA-DBS in randomized order. After 7 months follow-up (7MFU), programming parameters are not restricted until termination of the study at 12MFU.

### Study procedure

Patients are screened for study participation and implanted with DBS systems capable of current-controlled (mA) stimulation (Activa RC/PC, Medtronic or Vercise System, Boston Scientific) in the VIM and PSA bilaterally within 6 weeks of study enrollment. Both leads are implanted so that each lead has at least one contact inside the PSA, one inside the VIM, and one ”neutral contact” located on the intercommissural line (ICL, line connecting the anterior with the posterior commissure) in between the two target areas. Between implantation and randomization at 3MFU, this neutral contact is activated and adjusted for optimal tremor suppression with minimal side effects. This is done to ensure that all patients receive the same type of stimulation before entering the randomization phase of the study.

Only if implantation criteria are fulfilled, i.e., both leads are placed as described above and proper lead location is confirmed via stereotactic X-ray or postoperative CT scan, are patients included at 3MFU into the double-blinded, randomized phase of the study. Patients are randomized into two groups (group 1: VIM-DBS followed by PSA-DBS; group 2: PSA-DBS followed by VIM-DBS). The crossover is double-blinded (neither the patient nor the treating neurologist knows which area is stimulated in which period) and lasts for 4 months (2 months on each treatment). A programming visit (PV) is performed at the beginning of each period (at 3MFU and 5MFU) and the best clinical stimulation parameters of the respective contact are determined (i.e., best tremor suppression with no or tolerable side effects). The stepwise increase of current of the electrode contacts in the crossover phase yields a better understanding of the therapeutic windows (i.e., range between effect and side effect) of the two target areas. Double-blinding is also maintained during the programming visit, as adjustment of stimulation parameters is performed by an independent investigator (programmer) as instructed by the blinded rater. An evaluation visit (EV) is performed after each group (EV1 at 5MFU and EV2 at 7 MFU). As the effects and side effects are known to be immediate (although not systematically investigated for VIM-DBS), a longer washout phase between the two crossover phases is not necessary. After the crossover phase, the contact with the best effect and fewest side effects or more complex stimulation paradigms (such as double monopolar or bipolar configurations) can be freely chosen for further treatment until 12MFU.

For study question 1, the change from preoperative to postoperative TRS during 2 months of PSA stimulation will be analyzed. Therefore, the data will be pooled from the EV at 5MFU and 7MFU depending on randomization.

Patients without electrode placement according to the implantation criteria (see above) but with electrodes bilaterally located in the PSA will be stimulated in the PSA directly after implantation, evaluated at 3MFU and 12MFU, and will be excluded from the comparison between PSA and VIM. For the longitudinal PSA effect, 3MFU data from these patients will be pooled with 5MFU and 7MFU data from the randomization group, respectively.

During the entire study the patients are treated with current-controlled (mA) stimulation settings which allow a better comparability of the amount of current applied to the tissue in the crossover design. TRS will be video-recorded for blinded, external analysis of the primary endpoint. Patients not eligible for the study or patients who do not give informed consent for study participation will be treated according to our clinical standard.

### Population, screening, and recruitment

Patients with medication refractory ET will be screened and recruited at the Department of Neurology, Cologne University, Germany. A neurologist specialized in movement disorders (LT) will assess the patients’ eligibility for study participation.

### Inclusion and exclusion criteria

The inclusion and exclusion criteria of this study are in line with other ET-DBS studies as well as with the recommendations of the German DBS study group, which will help to ensure comparability [7]. Medication refractory ET is defined as at least two medical treatment attempts with at least two different medication groups (e.g., beta blockers, antiepileptic drugs) without a satisfying effect (either due to insufficient effect or side effects) for the patient.

In detail, the key inclusion criteria are:

1. Confirmed diagnosis of ET according to the Movement Disorder Society (MDS) consensus diagnostic criteria [26]
2. Age >18 years
3. Sufficient competence in the German language
4. At least two medication attempts without satisfactory tremor control
5. Capability to provide informed consent.

Exclusion criteria are:

1. Clinically relevant dementia that might interfere with the study
2. Clinically relevant psychiatric disorder that might interfere with the study
3. Surgical contraindication for bilateral DBS
4. Participation in another interventional trial
5. Brain atrophy (i.e., width of third ventricle >10 mm)
6. Preoperative and perioperative intake of anticoagulative medication.

### Outcome measures

The primary endpoint of this study is the reduction of tremor, measured via a reduction in TRS scores, due to PSA-DBS over 2 months compared to preoperative baseline. The TRS is a widely used and well-accepted scale for the clinical assessment of tremor and has been used in most controlled ET studies. The scale was first published in 1993 [24] and validated for ET in 2007 [27]. It consists of three parts: (A) tremor assessment, (B) task assessment, e.g., pouring water from one glass into another, writing, drawing spirals, and (C) a questionnaire assessing activities of daily living, e.g., eating soup with a spoon, social distress, hygiene; the different parts reflect the impairment due to tremor in different dimensions. The TRS has also been used as the primary endpoint measure in the retrospective analysis of Blomstedt et al. [13], which is the basis of power calculation for our study.

Secondary endpoints comprise general (SF-36 [25]) and disease-specific quality of life (QUEST [4], non-validated German version), depression (Beck Depression Inventory, BDI-II [28]), ataxia (International Cooperative Ataxia Rating Scale, ICARS [29]), and the patients’ and physicians’ subjective impressions on tremor severity and side effects (gait and speech) measured via visual analog scale (VAS). Stimulation-induced side effects are determined and recorded as adverse events throughout the study.

In addition, the amount of electric current applied during adjusted stimulation comprises a further endpoint. In case no difference in efficacy is seen between PSA and VIM stimulation in the crossover phase, it is particularly important to detect whether PSA stimulation requires less current on average and if the side effect profile differs in contrast to VIM stimulation. An acoustic speech sample is collected at each visit. The samples are analyzed by a phonetician and speech therapist as previously described [10, 30].

Together, these instruments provide a comprehensive assessment of subjective and objective treatment effects beyond mere motor effect.

### Sample size and power calculation

Study question 1 concerns the tremor reduction at 5/7MFU due to PSA-DBS compared with baseline. Blomstedt [13] measured a mean reduction in TRS from 46.2 at baseline to 18.7 one year later in 21 patients with ET treated with PSA-DBS, with standard deviations of 10.1 and 8.8, respectively.

For study question 2 comparing PSA-DBS with VIM-DBS, a power of 80 % at significance level 5 % can be attained for a difference of 9 points on the TRS, corresponding to one-third of the tremor reduction from baseline due to PSA-DBS, with a sample size of *n* = 12 (calculated using the program PS version 3.0.43). A correlation coefficient of 0.5 between the two post-stimulation values is assumed.

For study question 1, testing for tremor reduction from baseline under PSA stimulation, assuming the same values of significance level and power, using the paired *t* test, the sample size was calculated as *n* = 4. Alternatively, with the previously determined sample size of *n* = 12 a tremor reduction of 9.7 points could be detected. This calculation involved assuming that the baseline and post-stimulation values of a particular patient are positively correlated, with a correlation coefficient of 0.5.

In case of missing data on the tremor score due to loss to follow-up or withdrawal of consent, the patient does not contribute to the analysis. A 20 % safety margin was allowed for such cases, giving a target recruitment of 15 patients.

### Statistical analysis

For study question 1, the reduction of tremor due to PSA-DBS is calculated by subtracting the TRS score at baseline from the score obtained after the 2 months of continuous PSA stimulation (either 5MFU or 7MFU, depending on randomization).

As a secondary endpoint, the TRS score at 12 MFU under PSA stimulation is analyzed as a long-term parameter. Only some patients are stimulated in the PSA between 7MFU and 12MFU, since some patients might have chosen VIM stimulation after the randomization phase. Accordingly, a comparison between VIM and PSA stimulation at 12MFU might be possible (according to the group size).

For all other (secondary) endpoints evaluated for PSA-DBS, the difference between the score at 5/7MFU and the score at baseline is calculated.

For study question 2, the difference in tremor scores between PSA and VIM stimulation in each individual patient is calculated as the difference between TRS at 5MFU and 7MFU.

For all other (secondary) endpoints compared between PSA and VIM, the scores at 5MFU are compared with those at 7MFU, according to randomization group.

For study question 1, the mean reduction in tremor (TRS) due to PSA-DBS with its standard error and 95 % confidence interval is computed. The individual baseline and after-treatment values are tested against ”no reduction” using a paired *t* test. Similarly for SF-36, QUEST, and BDI-II, the mean change from baseline together with the corresponding 95 % confidence interval is calculated, and a paired *t* test is performed. A graphical display is used to examine the distribution of the data; in case of marked skewness or other non-conformity to test assumptions, the appropriate non-parametric tests are employed as a sensitivity analysis.

For study question 2, mean tremor at the end of the crossover period with PSA-DBS and VIM-DBS, respectively, is computed, with 95 % confidence intervals. A repeated measures analysis of variance, allowing for a period effect, is performed to quantify and test for a difference in mean tremor score between PSA and VIM in the crossover design. The mean difference between PSA and VIM with 95 % confidence interval is calculated from the model. Similarly for secondary outcomes, mean values under the two treatments are computed and compared using repeated measures analysis of variance.

All analyses are based on the intention-to-treat principle. Study question 1 is analyzed using the full analysis set including all randomized patients who begin PSA stimulation in the crossover period and who have a post-PSA stimulation evaluation. Study question 2 is analyzed using the crossover analysis set including all patients in the full analysis set who also begin VIM stimulation in the appropriate crossover period and who have a post-VIM stimulation evaluation. Additional analyses are performed on the per protocol set including all randomized patients who are eligible for the study according to all main inclusion/exclusion criteria and who are stimulated according to protocol in both crossover periods. Sensitivity analyses drop the requirement for post-stimulation examination data and instead impute any missing values (see below). The safety set includes all patients (randomized or not) who begin stimulation in any area (PSA, VIM, or ICL).

Since all patients included into the trial receive DBS electrodes, they are followed closely by our center for many years after the implantation. After completion of the study protocol, patients are seen at least every 6 months in our outpatient clinic. A careful evaluation and (if appropriate) adjustment of stimulation parameters usually leads to improvement of tremor suppression and quality of life. In the rare case of loss to follow-up, noncompliance, or revocation of study participation, the patient will be excluded from the per protocol analysis.

### Handling of missing data

If only one component of a multiple component score (such as TRS part A, B, or C; QUEST part A to E; or SF-36 item groups 3 to 11) is incomplete, the missing item value(s) will be imputed using the most frequent (mode) response to this item in the entire group at the corresponding time point.

If a value measured after DBS is completely missing, the patient will be excluded from the main analysis of the corresponding endpoint. However, in order to guard against bias, a sensitivity analysis is performed reincluding these patients and using multiple imputation methods.

### Randomization

Block randomization with random varying block length is employed in order to generate balanced allocations over the two crossover groups. Numbered, sealed envelopes are produced, and these are opened sequentially for each enrolled patient. Due to the small sample size and since a crossover design is employed, it is not considered useful to stratify the randomization.

### Implantation procedure

The general surgical procedure has been published in detail before. Patients are operated under local anesthesia with sedation (remifentanil and propofol).

In summary, after fixation of the stereotactic frame (CRW Stereotactic System, Integra Neurosciences, or Riechert-Mundinger frame), planning is performed on fused stereotactic CT/MRI images, with the Framelink (Medtronic Inc.) or STP 3.5 (Leibinger) planning station.

The trajectory is planned so that both VIM and PSA can be stimulated, with one electrode contact on the AC-PC level. Macrostimulation is performed in all patients. Microrecording is selected in a number of patients based on the surgeon’s preference. Implanted electrodes are quadripolar electrodes from Medtronic Inc. (model 3387 or 3389) or octopolar electrodes from Boston Scientific (model 616010).

Finally, the pulse generator (Activa PC, model 37601; Activa RC, model 37612, Medtronic, USA or Vercise, Boston Scientific, USA) is implanted subcutaneously in the infraclavicular or lateral abdominal region under general anesthesia.

### DBS programming

Directly after implantation and until 7MFU the activated electrode contact is determined by the study protocol (ICL, PSA, VIM or ICL, VIM, PSA), but the parameters can be freely chosen in terms of amplitude, frequency, and pulse width. However, all patients must be stimulated with constant current (mA), with a positive case and a single active cathode. In the crossover phase, at each programming visit, a monopolar review from 0 to 5 mA in 0.5-mA increments is performed. The amplitude with the best effect and fewest side effects is chosen for the final setting and is usually further adapted by the investigator during the programming visit before discharge. In general, a monopolar configuration with 60 μs and 130 Hz is chosen initially. In case a patient does not present a sufficient effect, the frequency and pulse width can be additionally modulated to improve the outcome.

If stimulation on a certain contact does not lead to a sufficient effect or intolerable side effects occur, and thus a change of the electrode contact or electrode configuration is inevitable, the corresponding crossover phase cannot be continued for ethical reasons. In this case the EV of the respective crossover phase must be performed immediately. After the end of the crossover phase, at 7MFU, the activated contact can be freely chosen.

### Blinding

During the crossover phase the study is performed in a double-blinded design. To ensure that neither the patient nor the physician is aware of the stimulation site (PSA or VIM), during the blinded crossover phase, each patient is seen by a blinded and an unblinded investigator (the latter usually being a study nurse). The unblinded study nurse performs all DBS programming during that phase. The physician decides according to clinical impression whether the stimulation parameters need to be modified (e.g., increase or decrease of amplitude or modulation of pulse width or frequency). The stimulation parameters and especially the activated contact (VIM or PSA) are documented in a separate file not accessible by the blinded physician. The blinded physician will be unblinded after all case report form (CRF) documentation of the crossover phase has been completed.

For the preoperative versus postoperative comparison of tremor suppression, all patients are videotaped and analyzed by an independent rater. The patients wear surgical caps before and after implantation, so that the rater cannot deduce the time point of data collection (i.e., preoperative versus postoperative) from, e.g., scars or short hair after implantation.

### Withdrawal from the study

In case of unexpected harm to the patient, insufficient compliance, or withdrawal of informed consent, a patient will promptly be excluded from further study treatment. In any case of premature withdrawal during the trial, the reasons for withdrawal must be documented in the CRF. The withdrawn subject will undergo a final examination (final visit) which must be documented.

### Safety

Implantation of electrodes in the PSA has so far not been associated with a higher risk for complications in these patients.

Adverse events (AEs)/serious adverse events (SAEs) are documented at the scheduled and unscheduled clinical visits. All incidents are reported to the regulatory authority. All safety-relevant events are promptly reported to the ethics committee and the safety monitor (Prof. J. Voges, Department of Stereotactic Neurosurgery, University of Magdeburg, Germany).

### Quality assurance/monitoring

Monitoring is performed by the Clinical Trials Centre Cologne (CTC Cologne). Monitors inspect the study center regularly to ensure implementation of the study protocol and high quality of documentation. An initiation visit, four regular visits, and a close-out visit are performed. Original source documents are reviewed for verification of data in the CRF. For each patient the presence of written consent is checked and the inclusion and exclusion criteria are controlled.

### Documentation

All data relevant to the trial are documented soon after measurement by the responsible investigator in the CRF supplied, with signature.

### Data management

IT infrastructure and data management are provided by the CTC Cologne. The data management system is based on commercial trial software and includes a database. The trial database was developed and validated before data entry based on standard operating procedures at the CTC Cologne. All changes made to the data are documented in an audit trail. The trial software has a user and role concept that can be adjusted on a trial-specific basis. The database is integrated into a general IT infrastructure and safety concept with a firewall and backup system. The data are backed up daily. After completion and cleaning of data, the database is locked and the data exported for statistical analysis.

The arrival of CRFs at the CTC Cologne is documented and the CRFs are checked for completeness. Data entry staff members enter the data into a validated trial database using independent double data entry, and the data entered are compared and reconciled afterwards. Plausibility checks are also conducted in the database. Discrepancies and implausible values are clarified in writing between the data manager and the trial site. The trial site has to answer these queries without unreasonable delay. Further details will be specified in the data management manual.

### Data protection

The provisions of data protection legislation will be observed. It is assured by the sponsor that all investigational materials and data will be pseudonymized in accordance with data protection legislation before scientific processing.

Trial subjects will be informed that their pseudonymized data will be passed on in accordance with provisions for documentation and notification pursuant to sections 12 and 13 of the Good Clinical Practice (GCP) regulations to the recipients described there. Subjects who do not agree that the information may be passed on in this way will not be enrolled into the trial.

## Discussion

This study investigates the effects and side effects of deep brain stimulation of the PSA and — with the help of the randomized crossover — contrasts the outcome with that of VIM stimulation. Although this monocentric study will primarily serve as a pilot study for a larger, multicentric trial, we expect direct findings to improve treatment of future patients with ET, e.g., through a better understanding of the side effect profiles of the two target areas.

So far, results on PSA versus VIM stimulation are conflicting, and it is unclear whether the higher efficacy of PSA stimulation compared to VIM stimulation is sustained in the clinical setting when the amount of current and the amount and severity of stimulation-induced side effects might vary between the two target areas. Theoretically, the higher efficacy of PSA stimulation over VIM stimulation might potentially be neutralized by the use of stronger current in the VIM group or more frequent side effects in the PSA group, the latter limiting acceptable program settings in that target area.

Therefore, first, this study was designed such that patients are stimulated with constant current and not constant voltage to facilitate direct comparison of the amount of current (independent of the impedance of the electrode contacts) applied to the target area in both groups. Secondly, our study strongly focuses on stimulation-induced side effects in both target areas. If our study detects a lower rate of side effects in one of the two groups, this could relativize the result on tremor suppression. In fact, it might be the therapeutic window, which comprises effect and side effect, and not the mere amount of tremor suppression that is crucial for preference of one or the other target area. Thirdly, the required sample size for demonstrating non-inferiority of PSA-DBS compared to VIM-DBS is the same as that calculated to demonstrate superiority if the non-inferiority margin is set at a difference of 9 Essential Tremor Rating Scale (ETRS) points in favor of VIM-DBS (with the same values of standard deviation, alpha, and power and assuming a true difference of zero). Therefore, our study has an acceptable power to show approximate non-inferiority for the case that tremor suppression is similar with both treatments, but side effects are reduced under PSA stimulation.

The main strength of this study is the randomization of the patients in the double-blinded crossover phase. Potential limitations of the study are the relatively small sample size and a possible period effect of the crossover. The results of this pilot study need to be confirmed in a larger trial. Like other trials investigating the effects of DBS for the treatment of ET, there are some other DBS-specific methodological limitations. It is commonly accepted that, due to the direct and strong effect of VIM-DBS in ET patients, a truly blinded analysis of the preoperative to the postoperative state is probably impossible [2]. To circumvent this bias, we have designed a double-blinded analysis for the crossover trial comparing VIM and PSA, videotaping the preoperative and postoperative tremor scores for blinded analysis by an independent rater. Assuming that VIM and PSA stimulations do not differ strongly and predictably in effectiveness or side effects, the randomized crossover comparison is thus effectively blinded.

### Trial status

At the time of submission, recruitment has not been completed.

## Abbreviations

CRF: Case report form
DBS: Deep brain stimulation
ET: Essential tremor
EV: Evaluation visit
MCP: Midcommissural point
MDS: Movement Disorder Society
MFU: Months follow-up
PSA: Posterior subthalamic area
PV: Programing visit
TRS: Tremor Rating Scale
VIM: Ventral intermediate nucleus
